# Marketing communications in health and medicine: perspectives from Willis-Knighton Health System

**DOI:** 10.1186/s12913-020-05598-4

**Published:** 2020-09-15

**Authors:** James K. Elrod, John L. Fortenberry

**Affiliations:** 1 Willis-Knighton Health System, 2600 Greenwood Road, Shreveport, LA 71103 USA; 2 LSU Shreveport, 1 University Place, Shreveport, LA 71115 USA

**Keywords:** Marketing communications, Promotion, Hospitals, Patients, Healthcare

## Abstract

**Background:**

Communications prowess is a key ingredient of productive healthcare delivery pursuits, with associated successes clearly positioning health and medical establishments for growth and prosperity. Many conveyance mechanisms are at the disposal of healthcare providers, permitting numerous opportunities for engaging current and prospective patients. For the best communicative outcomes, all must be considered when formulating marketing communications initiatives, with consideration first, of course, requiring that healthcare providers acquire an understanding of communications options and associated fundamentals.

**Discussion:**

In pursuing knowledge of communications options and related guidance, healthcare providers can benefit greatly by gaining operational perspectives from peer institutions. Over many decades, through scores of deployments, Willis-Knighton Health System has acquired significant communications prowess, prompting development of this special supplement in *BMC Health Services Research*, entitled “Marketing communications in health and medicine: perspectives from Willis-Knighton Health System,” with this particular article supplying a brief profile of the contents included in the associated supplement. Across the pages of the collection of articles contained in the supplement, attention specifically is directed toward the components of the marketing communications mix, foundational elements of communication, the patronage process, and the necessity for integrating marketing communications.

**Conclusions:**

Establishing an effective dialogue with current and prospective patients is an absolute necessity for healthcare organizations, warranting intensive efforts to master marketing communications. Given the imperative for excellence in marketing communications, it is hoped that the light shed by this supplement and its collection of articles will help healthcare providers better understand marketing communications and deploy associated initiatives successfully, affording greater patient engagement opportunities.

## Background

Health and medical establishments provide arguably the most essential services offered in any given community. From quality-of-life enhancements to life-saving interventions, the services provided by healthcare organizations are without parallel, making these entities key community assets. As facilitators of well-being and even life itself, health and medical institutions profoundly influence their respective marketplaces [1–3]. Indeed, healthy communities are productive communities, with exceptional community health emerging largely from the endeavors of those dedicated to supplying health and wellness services on behalf of populations. This, in turn, sets the stage for all-important economic opportunity which drives community development, growth, and viability, positively impacting individuals and institutions alike [2, 4–7]. But despite the skill of physicians, the magnitude of medical technologies, the compassion of nurses, or the benefit of any other associated investment, healthcare services possess very little impact potential unless they are communicated effectively to current and prospective patients [8–14].

Communications prowess is a key ingredient of productive healthcare delivery pursuits, with associated successes clearly positioning health and medical establishments for growth and prosperity while simultaneously yielding vital benefits for their various constituencies. As such, healthcare institutions must direct considerable attention toward shoring up communications capabilities, ensuring that audience engagement successes match patient care successes. Such pursuits invariably direct healthcare providers to the discipline of marketing, notably its marketing communications component, which focuses on engaging desired audiences in hopes of attracting patronage [1, 8–11]. Many conveyance mechanisms are at the disposal of healthcare providers, permitting numerous opportunities for engaging current and prospective patients [10, 11]. For the best communicative outcomes, all must be considered when formulating marketing communications initiatives, with consideration first, of course, requiring that healthcare providers acquire an understanding of communications options and associated fundamentals.

## Discussion

In pursuing knowledge of communications options and related guidance, healthcare providers can benefit greatly by gaining operational perspectives from peer institutions. Acquiring such insights can be difficult, given competitive sensitivities, but occasionally healthcare institutions are compelled to share knowledge in published accounts, motivated by desires to advance the state of knowledge of the healthcare industry. Encouraged, accordingly, Willis-Knighton Health System sought to contribute knowledge on the marketing communications front, a vital area with excellence being mandatory for successful healthcare operations and endeavors. Over many decades, through scores of deployments, the institution has acquired significant communications prowess [1, 15, 16], prompting development of this special supplement in *BMC Health Services Research*, entitled “Marketing communications in health and medicine: perspectives from Willis-Knighton Health System,” with this particular article supplying a brief profile of the contents included in the associated supplement.

The supplement’s first five articles profile the components of the marketing communications mix: advertising, personal selling, sales promotion, public relations, and direct marketing. “Advertising in health and medicine: using mass media to communicate with patients” provides a range of Willis-Knighton Health System’s insights and experiences garnered from extensive deployments of advertising, perhaps the best-known form of promotion. Conveying messages through paid use of mass media, advertising relies on television, radio, newspaper, billboard, and related mechanisms to transmit messages far and wide. The associated article discusses advertising’s interesting historical development in the health services industry, notes its current status as a mainstay communicative avenue, and profiles key motivations and considerations associated with its use.

“Personal selling in health and medicine: using sales agents to engage audiences” discusses the use of sales employees to personally deliver messages to individuals and institutions in desired markets. To many, the use of sales representatives by health services establishments seems somewhat foreign, but sales roles do exist in health and medicine, affording a critical communications capability. This particular article profiles Willis-Knighton Health System’s use of sales agents and discusses proper deployment strategies, yielding engaged and informed audiences. “Sales promotion in health and medicine: using incentives to stimulate patient interest and attention” spotlights the use of free samples, free trials, coupons, contests, loyalty programs, and the like by healthcare institutions as a means of reinforcing other components of the marketing communications mix, affording opportunities to better connect with patients. Among other things, Willis-Knighton Health System’s associated deployment strategies, including motivations and considerations in use, are outlined and explored.

“Public relations in health and medicine: using publicity and other unpaid promotional methods to engage audiences” profiles the communications avenue which traditionally has served as the primary method by which healthcare providers informed audiences of available offerings. This typically is achieved by preparing and submitting press releases to news media firms in hopes that they, in turn, will present given stories to their audiences. Public relations must be deployed carefully to realize communications goals, with this article presenting Willis-Knighton Health System’s strategies and perspectives. “Direct marketing in health and medicine: using direct mail, email marketing, and related communicative methods to engage patients” discusses the delivery of promotional messages directly to consumers. While some applications clearly have the potential to irritate consumers (e.g., junk mail in post boxes, spam in email inboxes), direct marketing can be deployed respectfully, yielding a helpful communications asset, with the associated article presenting viable strategies.

The next three articles of “Marketing communications in health and medicine: perspectives from Willis-Knighton Health System” shift attention toward complementary marketing communications facets. “Foundational elements of communication in health and medicine: avenues for strengthening the marketing communications mix” reminds readers that many things communicate on behalf of healthcare organizations, notably including the people employed by them, the places in which they deliver services, and the brands that represent them. As foundational elements of communication, these must be addressed prior to formulating the marketing communications mix, as they influence and impact an institution’s entire communicative potential, with this article profiling associated pathways. “Response hierarchy models and their application in health and medicine: understanding the hierarchy of effects” turns attention toward the patronage process by presenting models which describe the stages through which consumers pass on their way to becoming customers and patients of given healthcare establishments, permitting insights which can assist healthcare providers in their quests to hasten exchange and capture market share. “Integrated marketing communications: a strategic priority in health and medicine” discusses the need to cohesively assemble marketing communications, achieving harmony between and among components, permitting synergies which bolster institutional abilities to engage current and prospective patients.

The final article, “Reflecting on ‘Marketing communications in health and medicine: perspectives from Willis-Knighton Health System’: understanding the big picture,” concludes the supplement by taking the insights provided and presenting them in an operational framework, demonstrating the marketing communications process. This framework concisely summarizes the facets profiled in the associated articles, permitting readers to see how these pieces work in concert together in health and medical settings, providing a basic communications structure for advancing patient engagement initiatives, while also supplying a succinct summary of content, handily concluding the supplement.

## Conclusions

Given the imperative for excellence in marketing communications, health and medical providers must work intensively over the course of organizational life to develop associated prowess as a means of facilitating lasting patronage, yielding essential benefits for healthcare institutions, their customer populations, and their greater communities. Willis-Knighton Health System’s marketing communications insights and experiences, as profiled in this special supplement, supply helpful food for thought for advancing the patient engagement initiatives of most any healthcare institution, whether long established or newly initiated. The operational perspectives afforded nicely complement traditional textbook and trade publication portrayals, offering healthcare providers an opportunity to bolster their knowledge concerning one of the most vital practices associated with the pursuit and realization of institutional prosperity and its numerous mutual benefits.

## Data Availability

Not applicable.
